# Occurrence of *Hepatozoon *spp. in red foxes (*Vulpes vulpes*) in Romania

**DOI:** 10.1186/1756-3305-7-S1-O33

**Published:** 2014-04-01

**Authors:** MS Ilie, M Imre, K Imre, I Hotea, S Morariu, D Sorescu, S Andrei, C Mariş, G Dărăbuş

**Affiliations:** 1 Department of Parasitology and Parasitic Diseases, Faculty of Veterinary Medicine, BUASVM Timişoara, Romania; 2 Department of Forestry, Faculty of Horticulture and Forestry, BUASVM Timişoara, Romania

## 

*Hepatozoon *is an apicomplexan parasite of carnivores transmitted by the tick *Rhipicephalus sanguineus *trough ingestion. Infections in red foxes (*Vulpes vulpes*) have been recorded in several parts of the world, including European countries, with variable prevalence values.

The study aimed to investigate the occurrence of *Hepatozoon *spp. in red foxes originating from two counties (Arad and Timiş) of Western Romania.

Fresh/coagulated blood samples from 28 red foxes, killed in sanitary hunting during the routine rabies monitoring, were molecularly analyzed by a conventional PCR using the primers Hep F and Hep R for the presence of *Hepatozoon *spp.

Overall, 11/28 (39.2%) investigated samples contained the DNA of the parasite. Most of the positive samples were from Arad County (8/17), while in Timiş County only 3 out of 11 foxes tested were positive.

Occurrence of *Hepatozoon canis *was reported in Romania in dogs but investigations in wildlife were not performed.

The relatively high prevalence value of the infection recorded in the current survey can be related to the common occurrence of the brown dog tick (*Rhipicephalus sanguineus*) in the area, documented by previous surveys.

The high prevalence of the parasite among red foxes confirms the presence of sylvatic cycle, highlights the role of wildlife as reservoirs and potential vectors for the infection in the screened region and also the emerging of this pathogen agent in the country.

This is the first report of *Hepatozoon *spp. infection in red foxes from Romania. The findings support the global changing in vector borne diseases expansion.

## Funding

this study was financially supported by CNCS Grant TE_277_116/2010.

